# Characterization and reversal of Doxorubicin-mediated biphasic activation of ERK and persistent excitability in sensory neurons of *Aplysia californica*

**DOI:** 10.1038/s41598-017-04634-4

**Published:** 2017-07-03

**Authors:** Harini Lakshminarasimhan, Brittany L. Coughlin, Amber S. Darr, John H. Byrne

**Affiliations:** 1 Department of Neurobiology and Anatomy, W.M. Keck Center for the Neurobiology of Learning and Memory, McGovern Medical School at The University of Texas Health Science Center at Houston, 6431 Fannin St, Suite MSB 7.046, Houston, Texas 77030 USA; 2 McGovern Medical School at The University of Texas Health Science Center at Houston, 6431 Fannin St, Suite MSB 7.046, Houston, Texas 77030 USA; 3 Department of Physiology, Feinberg School of Medicine, Northwestern University, 303 E Superior Street, Chicago, 60611 Illinois USA; 4 The University of Texas MD Anderson Cancer Center UTHealth Graduate School of Biomedical Sciences, 6767 Bertner Ave, Mitchell Bldg. BSRB S3.8344, Houston, Texas 77030 USA

## Abstract

Doxorubicin (DOX), a common chemotherapeutic agent, impairs synaptic plasticity. DOX also causes a persistent increase in basal neuronal excitability, which occludes serotonin-induced enhanced excitability. Therefore, we sought to characterize and reverse DOX-induced physiological changes and modulation of molecules implicated in memory induction using sensory neurons from the marine mollusk *Aplysia californica*. DOX produced two mechanistically distinct phases of extracellular signal-regulated kinase (ERK) activation, an early and a late phase. Inhibition of MEK (mitogen-activated protein kinase (MAPK)/ERK kinase) after DOX treatment reversed the late ERK activation. MEK inhibition during treatment enhanced the late ERK activation possibly through prolonged downregulation of MAPK phosphatase-1 (MKP-1). Unexpectedly, the late ERK activation negatively correlated with excitability. MEK inhibition during DOX treatment simultaneously enhanced the late activation of ERK and blocked the increase in basal excitability. In summary, we report DOX-mediated biphasic activation of ERK and the reversal of the associated changes in neurons, a potential strategy for reversing the deleterious effects of DOX treatment.

## Introduction

Chemotherapy is associated with cognitive deficits including memory impairments in a subset of cancer survivors^1, 2^. Deficits in learning and memory can be persistent and may manifest during or long after chemotherapy in breast cancer patients^3, 4^. Doxorubicin (DOX), an anthracycline, is linked to deleterious side effects including cardiotoxicity and memory impairments in rodent models and humans^4–8^. The actions of DOX in other cell types may differ from its actions in tumor cells^9^. This is particularly evident in the nervous system, where DOX penetrates the blood-brain barrier at levels insufficient for anti-tumor action^10, 11^ but at concentrations sufficient to modulate synaptic plasticity and levels of molecules implicated in memory formation^12, 13^. Therefore, studies of the effects of DOX in neurons are warranted for characterization and determination of mechanisms underlying the memory deficits.

To examine the effects of DOX on neurons, the invertebrate model system *Aplysia* was chosen for its utility in studying long-term cellular and synaptic deficits caused by DOX treatment^12^. A single, brief treatment with DOX inhibits long-term synaptic facilitation (LTF) induced by pulsatile application of the neurotransmitter serotonin (5-HT), an *in vitro* analog of long-term memory (LTM) formation. DOX also facilitates long-term synaptic depression (LTD) mediated by the neuropeptide Phe-Met-Arg-Phe-NH_2_ (FMRFa) treatment. However, while DOX does not affect basal synaptic transmission, it enhances basal excitability of the presynaptic sensory neurons (SNs)^12^. Pulsatile 5-HT application results in long-term enhancement of excitability (LTEE)^12^, another correlate of LTM formation^14^. It is thus logical to expect that aberrant changes in basal excitability will interact with learning-associated changes in excitability. This hypothesis is supported by the observation that DOX treatment results in enhanced basal excitability and prevents any additional 5-HT-mediated increases in excitability^12^, suggesting that learning-associated changes in excitability may be blocked by DOX. Thus, the basal activation of the ERK pathway and the enhanced basal excitability by DOX may occlude any further changes required for learning and memory formation. In addition, reducing aberrant neuronal activity alleviates DOX-mediated deleterious effects^15^. Thus, reversing the increased excitability may be a key first step towards restoring normal 5-HT-mediated increases in LTEE in the presence of DOX, and may thus be helpful for mitigating side effects of DOX treatment.

DOX treatment also leads to the activation of two mitogen-activated protein kinases (MAPK), extracellular signal-regulated kinase (ERK) and p38 MAPK. ERK and p38 MAPK exert opposing actions on synaptic plasticity. In both vertebrates and invertebrates, ERK promotes synaptic facilitation, and p38 MAPK mediates synaptic depression^16–26^. FMRFa-mediated activation of p38 MAPK prevents basal activation of ERK^27^, possibly through activation of a protein phosphatase^28, 29^. DOX-mediated impairment of LTF is rescued by inhibition of p38 MAPK^12^, suggesting that the DOX-mediated activation of p38 MAPK may dominate over ERK and contribute to the deficits in synaptic facilitation and enhancement of synaptic depression. Thus, it is of interest to determine the time-course and persistence of activation of both ERK and p38 MAPK. Differences in the onset or duration of activation of the two kinases may shed light on why the effects of p38 MAPK seem to dominate over those of ERK in response to DOX treatment.

In addition to the role of ERK in modulating synaptic strength, ERK enhances neuronal excitability^30–34^. Activation of ERK is sufficient to induce long-lasting changes in excitability of *Aplysia* SNs^34^ and is required for enhanced long-term excitability by transforming growth factor β-1 (TGFβ-1)^31^. This latter study showed that even a transient enhancement of ERK activity was sufficient to cause enhanced excitability for at least 24 h^31^. The role of p38 MAPK in excitability is less clear. p38 MAPK has been implicated in suppression of excitability in pyramidal neurons^35, 36^ but also in the hyperexcitability of neurons under certain pathological conditions^37–39^. Enhancement of basal excitability of the presynaptic cell by DOX accompanied by deficits in synaptic facilitation suggest divergent roles of kinase activation in mediating neuronal excitability and synaptic plasticity. Therefore, it is of particular interest to determine if the DOX-induced increase in basal neuronal excitability depends upon either or both ERK and p38 MAPK activation.

DOX also leads to decreased expression of MAPK phosphatase-1 (MKP-1) in SNs^12^. MKP-1-mediated dephosphorylation of active MAPK plays a critical role in the feedback control of MAPK cascades in a variety of cellular processes^40^. For example, activation of ERK by pulsatile application of 5-HT to SNs is blocked by pre-treatment with purified MKP-1^41^. MAPK activation is a reflection of the balance between MEK-mediated phosphorylation and phosphatase-mediated dephosphorylation^42–45^. Thus, understanding the temporal modulation of MKP-1 expression following DOX treatment and the upstream contribution of MEK to kinase activation will shed light on the mechanism of MAPK activation in response to DOX treatment. These results will help in determining a method for reversing DOX-induced changes to kinase activation.

## Results

### Time-course of DOX-induced activation of ERK and p38 MAPK in SNs during DOX treatment

**Figure 1:**
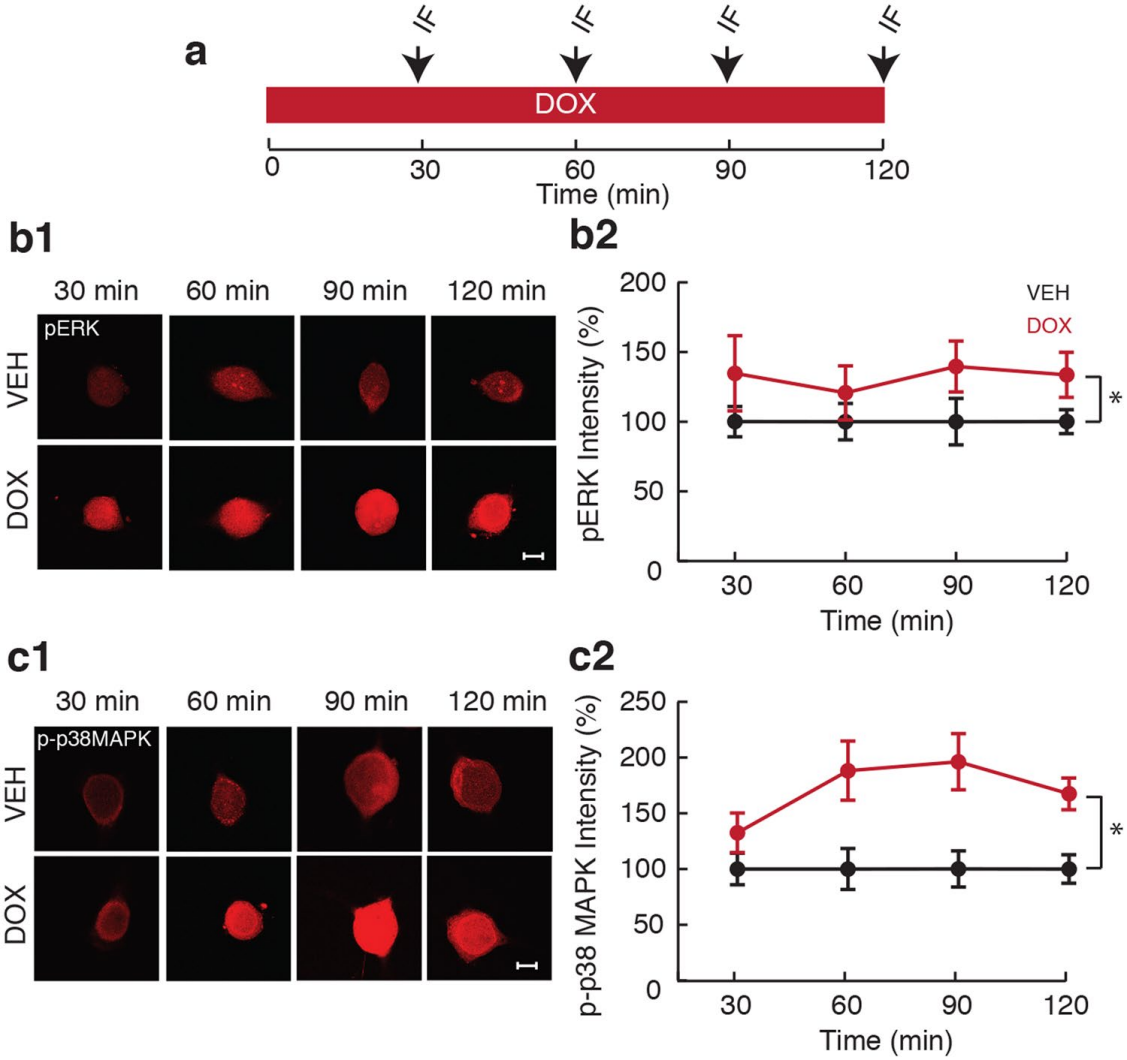
Effects of duration of DOX treatment on ERK and p38 MAPK activation in SNs. (**a**) Protocol for DOX application of various durations and measurement of kinase phosphorylation. Arrows represent fixation times followed by immunofluorescence staining (IF). (**b1**) Representative confocal images of pERK staining in SNs treated with DOX for 30, 60, 90, and 120 min. Scale bar, 20 µm. (**b2**) Summary data. Fluorescence intensity is indicated as percentage change compared to Veh. (**c1**) Representative confocal images of p-p38 MAPK immunofluorescence in SNs treated with DOX for 30, 60, 90, and 120 min. Scale bar, 20 µm. (**c2**) Summary data. DOX treatment resulted in rapid activation of ERK and p38 MAPK which lasted throughout the duration of treatment. Data are plotted as mean ± SEM; *represents p ≤ 0.05.

We used immunofluorescence to measure levels of activated (phosphorylated) ERK (pERK) and p38 MAPK (p-p38 MAPK) in isolated SNs during DOX treatment. The temporal profiles of pERK and p-p38 MAPK resulting from varying durations of DOX treatment (2.5 µM for 30, 60, 90, and 120 min; Fig. 1a) were examined to determine whether the dynamics of activation of these kinases differ. DOX treatment led to an increase in pERK to 134.7 ± 27% (*n* = 7) at 30 min, 120.7 ± 19.4% (*n* = 8) at 60 min, 139.6 ± 18.3% (*n* = 8) at 90 min, and 133.6 ± 16.2% (*n* = 8) at 120 min (Fig. 1b) compared to vehicle (Veh) treatment (100 ± 10.9% (*n* = 7) at 30 min, 100 ± 13.0% (*n* = 8) at 60 min, 100 ± 16.7% (*n* = 8) at 90 min, and 100 ± 8.6% (*n* = 8) at 120 min). A two-way ANOVA revealed an overall effect of treatment (F_(1,54)_ = 7.21; p = 0.01) but no effect of time (F_(3,54)_ = 0.12; p = 0.95) or interaction of treatment with time (F_(3,54)_ = 0.12; p = 0.95). Therefore, DOX led to rapid activation of ERK that persisted for the duration of treatment.

Similarly, DOX led to an increase in p-p38 MAPK to 132.4 ± 17.7% (*n* = 6) at 30 min, 188.1 ± 26.5% (*n* = 7) at 60 min, 196.2 ± 25.2% (*n* = 8) at 90 min, and 167.4 ± 14.2% (*n* = 7) at 120 min compared to Veh (100 ± 14.1% (*n* = 6) at 30 min, 100 ± 18.3% (*n* = 7) at 60 min, 100 ± 16.2% (*n* = 8) at 90 min, and 100 ± 12.7% (*n* = 7) at 120 min) (Fig. 1c). A two-way ANOVA revealed an overall effect of treatment (F_(1,48)_ = 26.94; p = 0.000004) but no effect of time (F_(3,48)_ = 1.03; p = 0.39) or interaction of treatment with time (F_(3,48)_ = 1.03; p = 0.39). Thus, similar to its effects on ERK, DOX led to rapid and sustained activation of p38 MAPK. We next determined the persistence of pERK and p-p38 MAPK following removal of DOX.

### DOX induced biphasic activation of ERK after the end of DOX treatment

**Figure 2:**
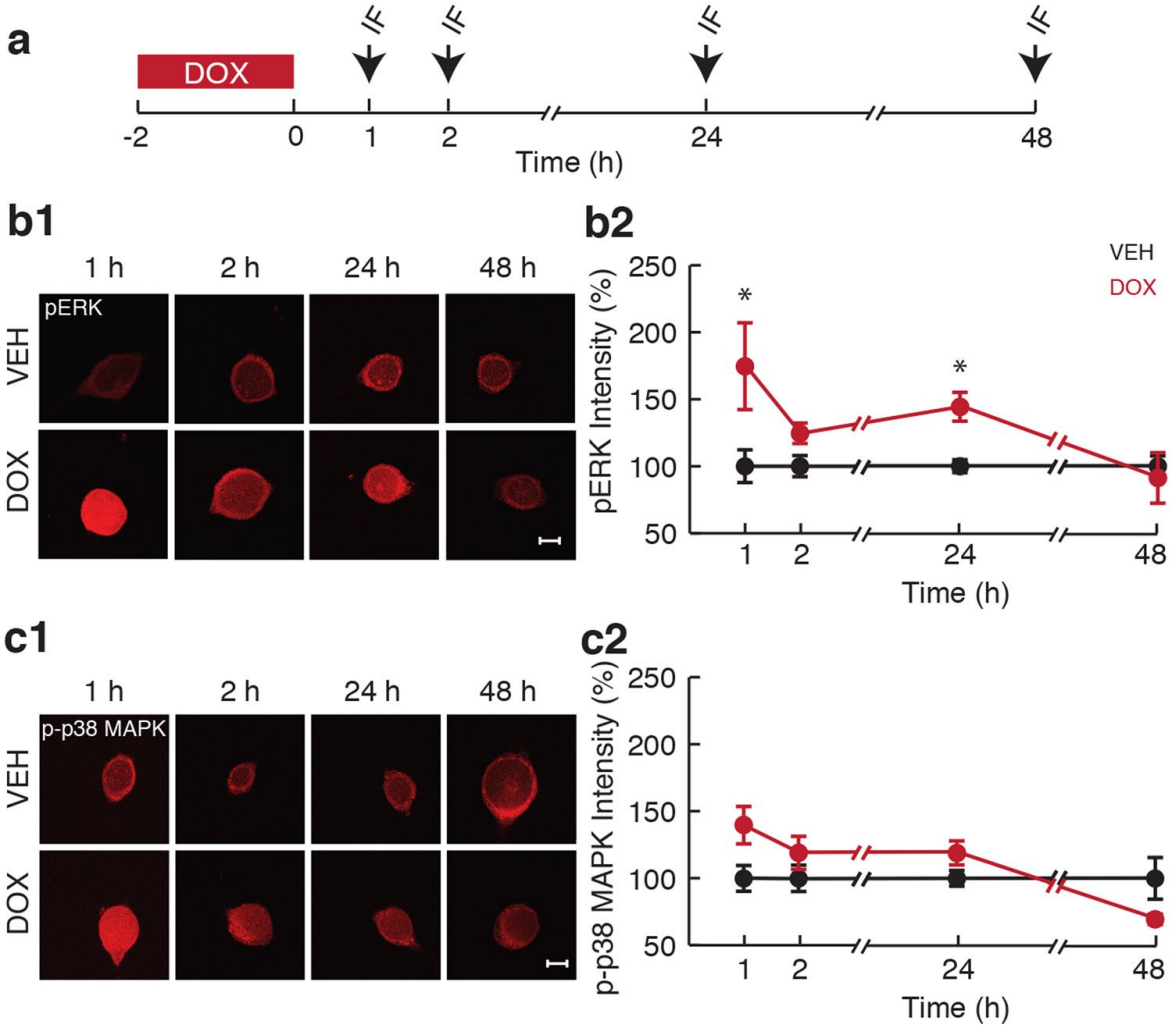
Persistent activation of ERK but not p38 MAPK in response to DOX treatment. (**a**) Protocol for application of DOX and measurement of persistent kinase phosphorylation in SNs. Arrows represent fixation times and subsequent IF staining. (**b1**) Representative confocal images of pERK staining in SNs 1, 2, 24, and 48 h after the end of DOX treatment. Scale bar, 20 µm. (**b2**) Summary data. (**c1**) Representative confocal images of p-p38 MAPK immunofluorescence in SNs 1, 2, 24, and 48 h after the end of DOX treatment. Scale bar, 20 µm. (**c2**) Summary data. DOX treatment resulted in an early and a late phase of ERK activation. The increase in p-p38 MAPK levels was reversed within 2 h of treatment cessation. Data are plotted as mean ± SEM; *represents p ≤ 0.05.

Because both ERK and p38 MAPK showed enhanced activation levels during DOX treatment (Fig. 1), the question arose as to how long their activation levels remain elevated after removal of DOX. Therefore, the persistence of both pERK and p-p38 MAPK was examined 1, 2, 24, and 48 h after the end of DOX treatment (2.5 µM, 2 h) (Fig. 2a). DOX exhibited differential effects on activation of these two kinases. pERK increased to 174.6 ± 32.4% (*n* = 8) 1 h after treatment, 119.2 ± 7.6% (*n* = 9) at 2 h, 144.3 ± 10.7% (*n* = 10) at 24 h, but decreased to 91.2 ± 18.8% (*n* = 8) at 48 h in comparison to Veh (100 ± 12.2% (*n* = 8) 1 h after treatment, 100 ± 7.9% (*n* = 9) at 2 h, 100 ± 4.9% (*n* = 10) at 24 h, and 100 ± 7.7% (*n* = 8) at 48 h) (Fig. 2b). A two-way ANOVA revealed a significant effect of treatment (F_(1,62)_ = 10.93; p = 0.002), time (F_(3,62)_ = 2.76; p = 0.05) and interaction of treatment with time (F_(3,62)_ = 2.76; p = 0.05). Pairwise *post-hoc* comparisons revealed significant differences in pERK levels between DOX- and Veh-treated groups at 1 h (p = 0.0001) and 24 h (p = 0.023), but not at 2 h (p = 0.22) or 48 h (p = 0.68). In summary, DOX led to biphasic activation of ERK characterized by an early and a late phase following treatment.

The effect of DOX treatment on p-p38 MAPK was transient. Compared to Veh (100 ± 9.7%, *n* = 8), DOX induced p-p38 MAPK levels that reached 139.7 ± 13.9% (*n* = 8) 1 h after the end of treatment, although this effect was not statistically significant. There was no apparent increase at later times (108.1 ± 12.4% at 2 h, *n* = 9; 119 ± 8.9% at 24 h, *n* = 10) when compared to Veh (100 ± 9.8% at 2 h, *n* = 9; 100 ± 5.8% at 24 h, *n* = 10). Indeed, p-p38 MAPK decreased at 48 h to 69.2 ± 3.9% (*n* = 9) when compared to Veh (100 ± 15.5% at 48 h, *n* = 9) (Fig. 2c). A two-way ANOVA revealed no effect of treatment (F_(1,64)_ = 2.5; p = 0.12) but a significant effect of time (F_(3,64)_ = 3.9; p = 0.01) and interaction of treatment with time (F_(3,64)_ = 3.9; p = 0.01), suggesting that DOX-induced activation of p38 MAPK decreases over time.

In summary, both ERK and p38 MAPK were activated rapidly (Fig. 1), but only ERK exhibited a late phase of activation (Fig. 2). DOX-induced activation of ERK may be due to decreased expression of the MAPK phosphatase, MKP-1^46, 47^ and/or activation of the MEK pathway^48^. We first examined levels of MKP-1 protein.

### DOX caused a transient decrease in levels of MKP-1 protein

**Figure 3:**
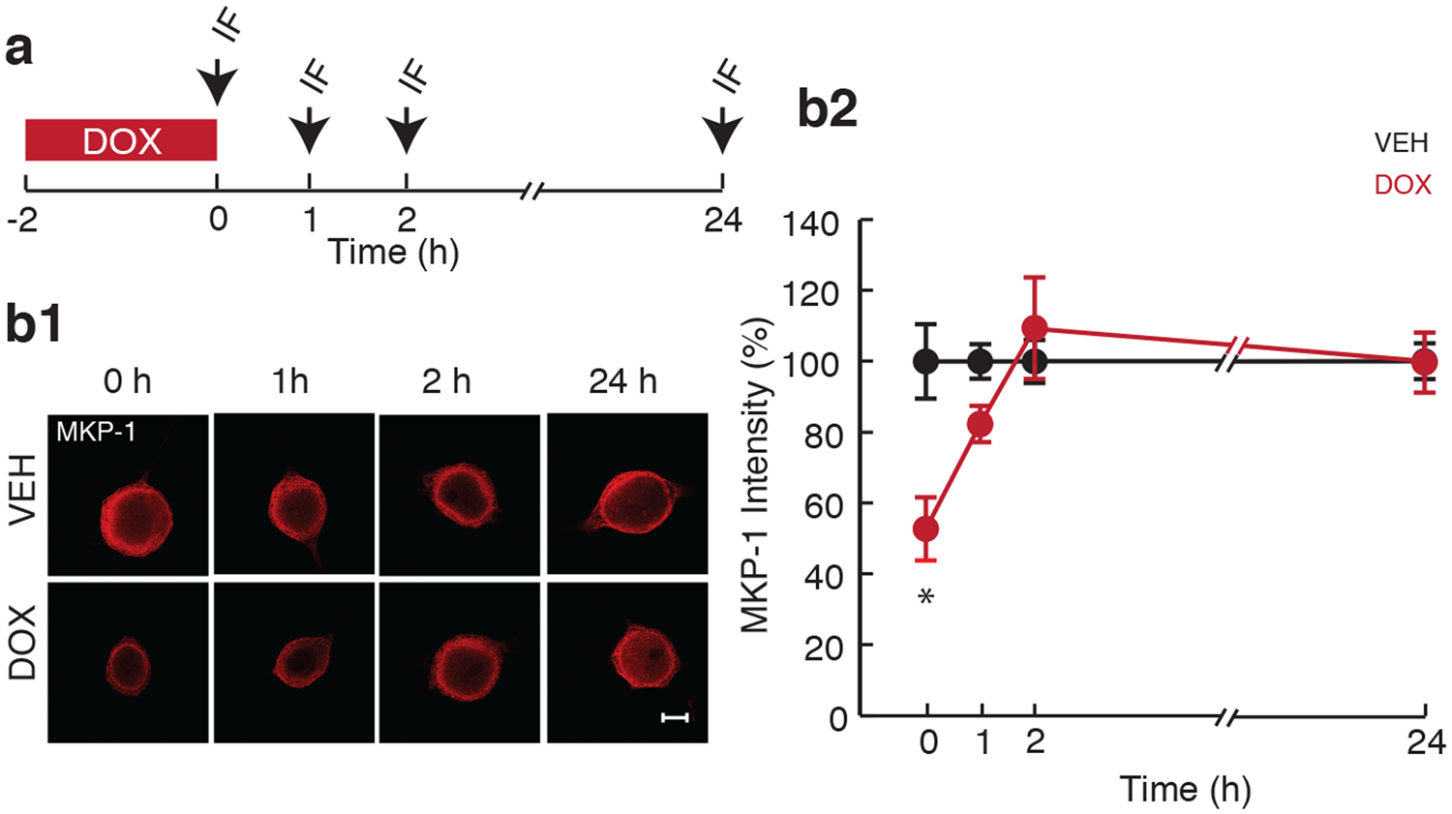
Transient decrease in expression of MKP-1 in response to DOX treatment. (**a**) Protocol for DOX application and measurement of MKP-1 expression levels. Arrows represent fixation times and subsequent IF staining. (**b1**) Representative confocal images of MKP-1 staining in SNs 0, 1, 2, and 24 h after the end of DOX treatment. Scale bar, 20 µm. (**b2**) Summary data. The decrease in levels of MKP-1 was reversed within 2 h after the end of treatment. Data are plotted as mean ± SEM; *represents p ≤ 0.05.

DOX downregulates MKP-1 in a ubiquitin-proteasome-dependent manner^46, 47^. To determine whether a decrease in MKP-1 protein could account for the DOX–mediated activation of ERK, we measured levels of MKP-1 protein at multiple times after DOX application to SNs using immunofluorescence (Fig. 3a). MKP-1 levels decreased to 52.8 ± 8.9% (*n* = 4) immediately after treatment (0 h) compared to Veh (100 ± 10.5%, *n* = 6) and remained depressed 1 h after the end of treatment (82.4 ± 4.7%, *n* = 9) compared to Veh (100 ± 7.2%, *n* = 10). By 2 h after the end of the treatment, MKP-1 levels returned to baseline (109.4 ± 12%, *n* = 9) compared to Veh (100 ± 8.6%, *n* = 10), and remained at baseline (99.6 ± 10.6%, *n* = 10) compared to Veh (100 ± 8.1%, *n* = 11) at 24-h post treatment (Fig. 3b). A two-way ANOVA revealed a significant effect of treatment (F_(1,60)_ = 4.1; p = 0.047) and trends toward a main effect of time (F_(3,60)_ = 2.6; p = 0.057) and interaction of treatment with time (F_(3,60)_ = 2.6; p = 0.057). Pairwise *post-hoc* comparisons revealed a significant difference between MKP-1 levels in DOX- and Veh-treated groups at 0 h (p = 0.009). In summary, DOX led to a transient decrease in levels of MKP-1 protein. Both ERK and p38 MAPK are substrates for MKP-1^40^, and the initial increase in activation of the two kinases mirrors the transient decrease in MKP-1 expression. The reduction in levels of MKP-1 may contribute to the increased levels of pERK and p-p38 MAPK immediately after DOX. Furthermore, MKP-1 levels returned to baseline by 2 h, at which point neither pERK nor p-p38 MAPK is significantly different between DOX- and Veh-treated cells (Fig. 2b,c). 24 h after DOX treatment, at a time when MKP-1 levels had long returned to baseline, a late phase of ERK activation was observed (Fig. 2b), suggesting that this late activation of ERK occurs by some mechanism other than MKP-1 downregulation. Candidate mechanisms investigated include: 1) upstream activation via MEK and/or 2) crosstalk between ERK and p38 MAPK pathways. We aimed to determine the contribution of MEK to both the early and late phases of ERK activation.

### Activation of ERK immediately after DOX treatment was MEK-independent

**Figure 4:**
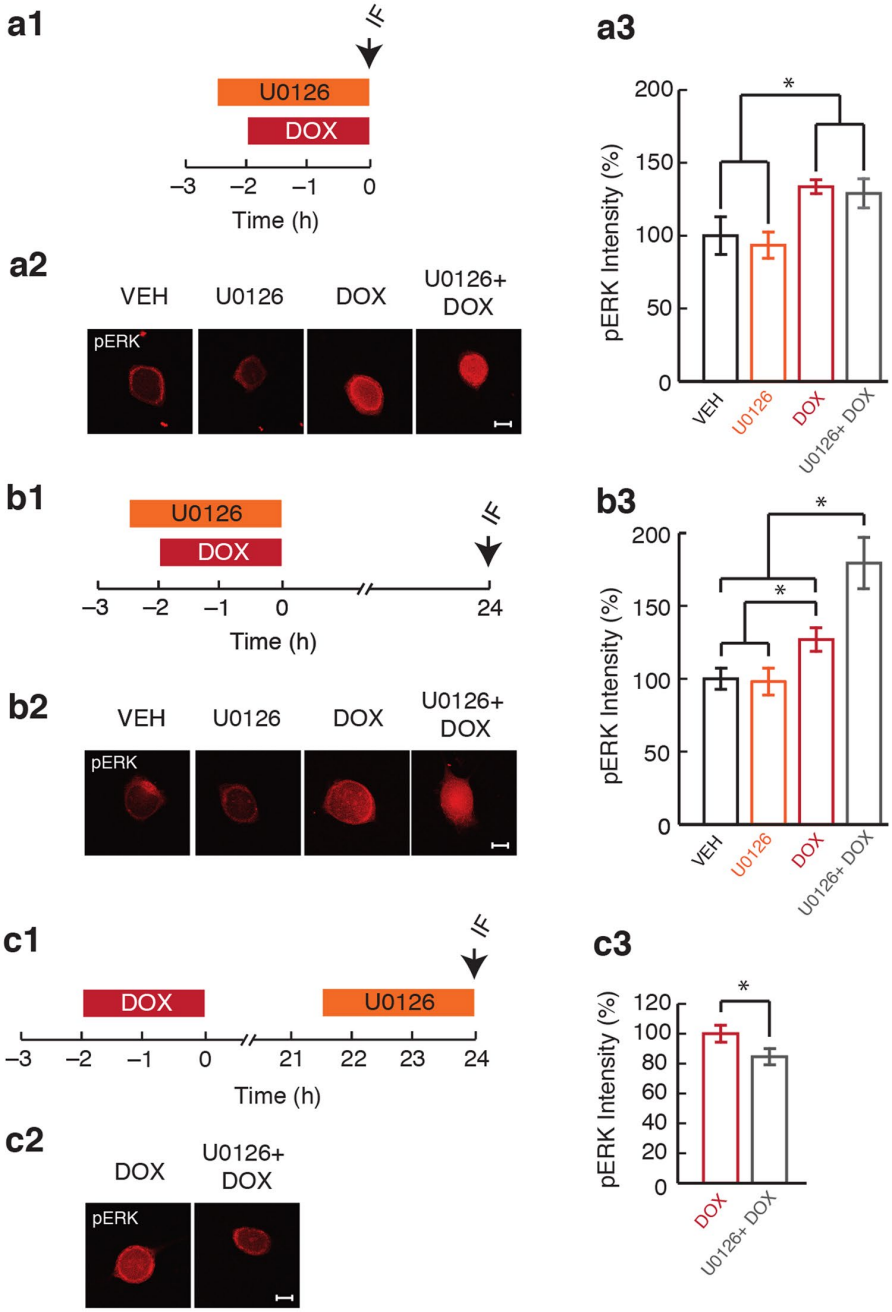
The late phase of pERK was potentiated by inhibition of MEK during DOX treatment but attenuated by inhibition of MEK after DOX treatment. (**a1**) Protocol for application of U0126 and DOX followed by measurement of pERK immediately after the end of treatment. Arrow represents fixation time and subsequent IF staining. (**a2**) Representative confocal images of pERK staining in SNs immediately after the end of DOX treatment. Scale bar, 20 µm. (**a3**) Summary data. (**b1**) Protocol for application of U0126 and DOX followed by measurement of pERK 24 h after the end of treatment. Arrow represents fixation time and subsequent IF staining. (**b2**) Representative confocal images of pERK staining in SNs 24 h after the end of DOX treatment. Scale bar, 20 µm. (**b3**) Summary data. (**c1**) Protocol for application of U0126 after the end of DOX application followed by measurement of pERK 24 h after the end of DOX treatment. Arrow represents fixation time and subsequent IF staining. (**c2**) Representative confocal images of pERK staining in SNs 24 h after the end of DOX treatment. Scale bar, 20 µm. (**c3**) Summary data. U0126 application after DOX treatment attenuated pERK levels at 24 h. Data are plotted as mean ± SEM; *represents p ≤ 0.05. See also Supplementary Fig. S1.

DOX enhances activation of ERK through upstream kinases or through downregulation of phosphatases^46–48^. We investigated the contribution of MEK to the early phase of ERK activation, immediately after the end of DOX treatment, at a time when MKP-1 is downregulated. The MEK inhibitor U0126 was applied during DOX treatment to determine the relative contribution of MEK to ERK activation (Fig. 4a1). Four groups were included in the experiment: (1) Veh; (2) U0126; (3) DOX; and (4) U0126 + DOX. As shown previously (Fig. 1b), DOX treatment led to an immediate increase in pERK (133.6 ± 4.7%, *n* = 10) compared to Veh (Fig. 4a). However, the increase in pERK was not blocked by pretreatment with U0126. U0126 + DOX led to an increase in pERK comparable to DOX alone (129.0 ± 10%, *n* = 10). Treatment with U0126 alone led to a slight decrease in pERK levels (93.4 ± 9.0%, *n* = 10) (Fig. 4a). A two-way ANOVA revealed a significant main effect of DOX (F_(1,36)_ = 12.9; p = 0.0001) as well as no basal effect of U0126 (F_(1,36)_ = 0.3; p = 0.57). The DOX-induced increase in pERK was not blocked by U0126 (DOX x U0126; F_(1,36)_ = 0.01; p = 0.92).

Immediately after the end of DOX treatment, MKP-1 protein levels decreased but returned to baseline 2 h after the end of treatment (Fig. 3). Inhibition of MEK during DOX treatment did not affect pERK levels immediately after the end of treatment (Fig. 4a). Together, these results suggest that the contribution of upstream MEK activation to ERK activation immediately following removal of DOX is minimal. It is likely that the coincident decrease in expression of MKP-1 accounts for this early phase of ERK activation.

### Late ERK activation was enhanced by MEK inhibition during DOX treatment

Because DOX caused biphasic ERK activation, it is possible that MEK inhibition during DOX treatment has differential effects on the early and late ERK activation. To determine whether the late ERK activation is affected by MEK activation during DOX treatment, U0126 was applied before and during DOX treatment and pERK levels were measured 24 h later (Fig. 4b). The same four groups were included in the experiment: (1) Veh; (2) U0126; (3) DOX; and (4) U0126 + DOX. As observed previously, DOX treatment led to a late increase in pERK levels (136.3 ± 10.4%, *n* = 17) compared to the Veh group (100 ± 10.4%, *n* = 17). However, the combination of U0126 and DOX led to an even greater increase in pERK (180.7 ± 17.8%, *n* = 15). Treatment with U0126 alone led to a slight decrease in pERK levels to 98.7 ± 9.2% (*n* = 15) (Fig. 4b). A two-way ANOVA revealed a significant main effect of DOX (F_(1,60)_ = 26.7; p = 0.0001) but no effect of U0126 (F_(1,60)_ = 3.6; p = 0.06). The DOX-induced late increase in pERK was enhanced by application of U0126 during DOX treatment (DOX x U0126; F_(1,60)_ = 4.0; p = 0.05). MEK inhibition during DOX treatment did not affect the early ERK activation (Fig. 4a), but enhanced the late ERK activation (Fig. 4b). These results suggest that the two phases are mechanistically distinct, and the late ERK activation can be modulated independent of the early ERK activation. The delayed effects of MEK inhibition on DOX-induced pERK levels indicate that early MEK activation by DOX contributes to the second phase of ERK activation. The finding that immediate ERK activation is not affected by application of the MEK inhibitor (Fig. 4a) suggests that DOX-induced activation of MEK is suppressed by the end of a 2-h treatment with DOX, possibly due to the accumulation of p38 MAPK which can inhibit the MEK pathway^27^.

Pertaining to the late activation of ERK, positive feedback loops within, or containing elements of, the ERK signaling cascade have been cited as plausible mechanisms underlying persistent ERK activation post-stimulus^49, 50^. Furthermore, crosstalk among MAPK families has been repeatedly documented^49, 50^. Interactions between DOX-mediated activation of p38 MAPK and ERK may contribute to the late phase of ERK activation as the late activation of ERK by DOX may depend on the initial increase in p38 MAPK activation. However, inhibition of p38 MAPK activity during DOX treatment did not affect the late phase of ERK activation (Supplementary Fig. S1).

### Late ERK activation was reversed by inhibition of MEK after the end of DOX treatment

In response to DOX treatment, late ERK activation (24 h after the end of treatment), may depend on MEK given that MKP-1 expression has returned to baseline (Fig. 3b). To assess the contribution of MEK to the DOX-induced late ERK activation, SNs were treated with U0126 for 2.5 h prior to the 24-h measurement of pERK (Fig. 4c). This experiment employed two groups: (1) DOX + Veh; and (2) DOX + U0126 (Fig. 4c). Earlier experiments revealed that U0126 had no basal effect on pERK in comparison to Veh (Fig. 4a,b). In comparison to the DOX + Veh group (100 ± 5.7%, *n* = 12), the DOX + U0126 group (84.5 ± 5.7%, *n* = 12) showed a significant decrease in pERK (t_(11)_ = 2.515; p = 0.029). Thus, delayed inhibition of MEK, at a time when MKP-1 levels returned to baseline, attenuated the late ERK activation. These results suggest that DOX-induced activation of ERK consists of two mechanistically distinct phases, an early MEK-independent phase and a late MEK-dependent phase.

### Inhibition of MEK during DOX treatment resulted in long-lasting downregulation of MKP-1

**Figure 5:**
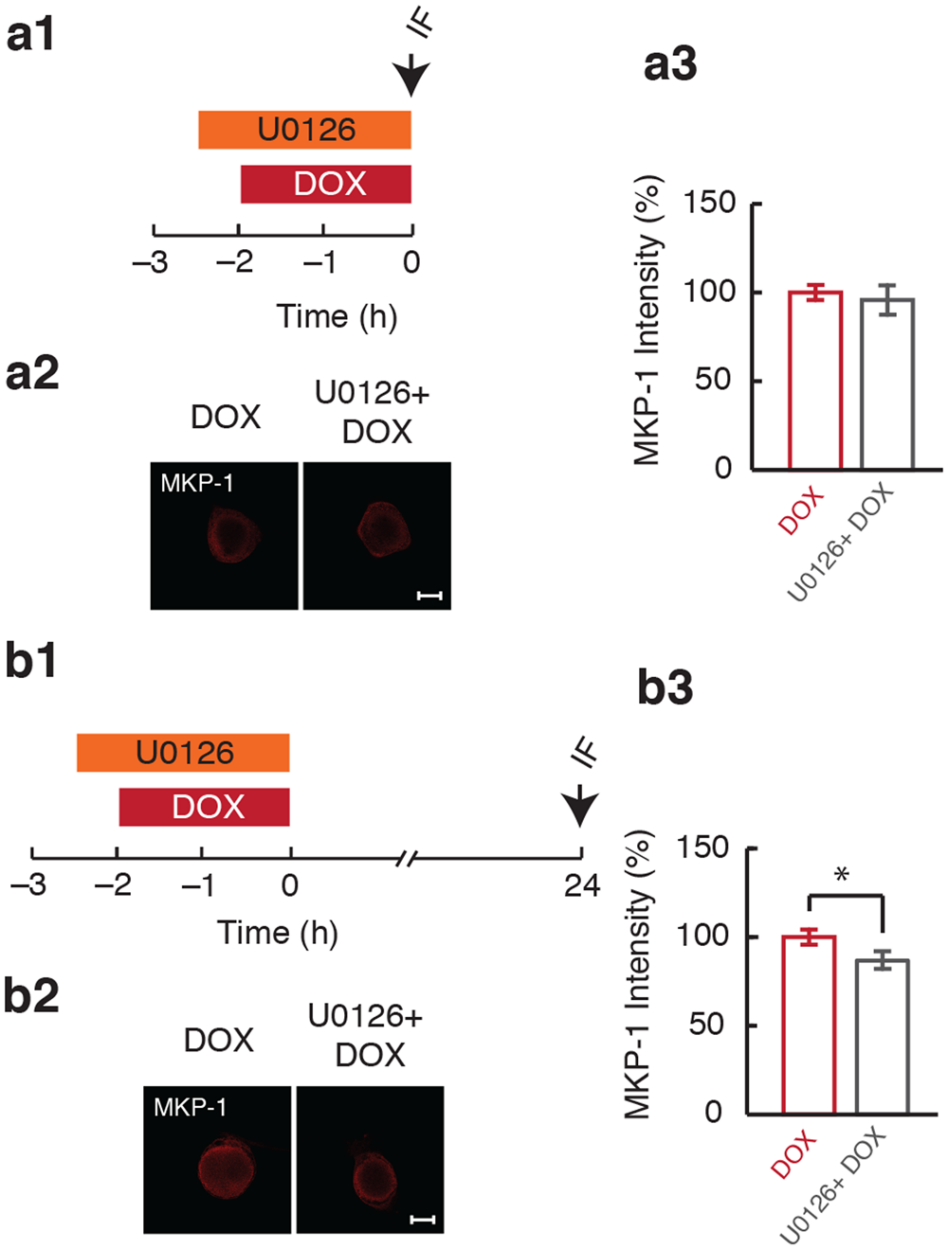
Inhibition of MEK caused a persistent decrease in MKP-1 levels following DOX treatment. (**a1**) Protocol for application of U0126 and DOX followed by measurement of MKP-1 expression immediately after the end of DOX treatment. Arrow represents fixation time and subsequent IF staining. (**a2**) Representative confocal images of MKP-1 staining in SNs immediately after the end of DOX treatment. Scale bar, 20 µm. (**a3**) Summary data. (**b1**) Protocol for application of U0126 and DOX followed by measurement of MKP-1 expression 24 h after the end of treatment. Arrow represents fixation time and subsequent IF staining. (**b2**) Representative confocal images of MKP-1 staining in SNs 24 h after the end of DOX treatment. Scale bar, 20 µm. (**b3**) Summary data. Application of U0126 caused a decrease in MKP-1 expression 24 h after DOX treatment. Data are plotted as mean ± SEM; *represents p ≤ 0.05.

DOX caused a transient decrease in MKP-1 protein levels (Fig. 3). To explore a possible mechanism by which MEK inhibition facilitates late ERK activation (Fig. 4b), we determined how MEK inhibition affects levels of MKP-1 protein well after the removal of DOX using immunofluorescence (Fig. 5a1 and b1). It is possible that inhibition of MEK prolongs the decrease in expression of MKP-1 protein given the established role of the MEK-ERK pathway in inducing MKP-1, which can serve as a feedback inhibition mechanism to limit ERK activation^51–54^. Therefore, inhibition of MEK may decrease MKP-1 expression prolonging the DOX-induced decrease in MKP-1, and thereby contribute to the enhancement of DOX-induced late ERK activation (Fig. 4b). To test this hypothesis, the effects of U0126 treatment on DOX-mediated changes in levels of MKP-1 protein immediately and 24 h after the end of treatment were examined.

Immediately after the end of treatment (0 h), at a time when MKP-1 levels were decreased by DOX alone (Fig. 3), both the DOX (100 ± 4.2%, *n* = 8) and U0126 + DOX groups (95.7 ± 8.2%, *n* = 8) showed comparable levels of MKP-1 protein (t_(7)_ = 0.4; p = 0.33) (Fig. 5a). However, 24 h after the end of treatment, at a time when DOX alone had no effect on MKP-1 (Fig. 3), the U0126 + DOX group (86 ± 4.6%, *n = 9*) showed a significant decrease in levels of MKP-1 protein (t_(8)_ = 1.9; p = 0.05) in comparison to DOX alone (100 ± 4.3%, *n* = 9) (Fig. 5b). Thus, inhibition of MEK during DOX treatment prolongs downregulation of MKP-1, which may, in combination with delayed or persistent MEK activation (Fig. 4c), contribute to enhancement of the late ERK activation (Fig. 4b).

### Long-lasting enhancement of excitability by DOX depended on MEK activity

**Figure 6:**
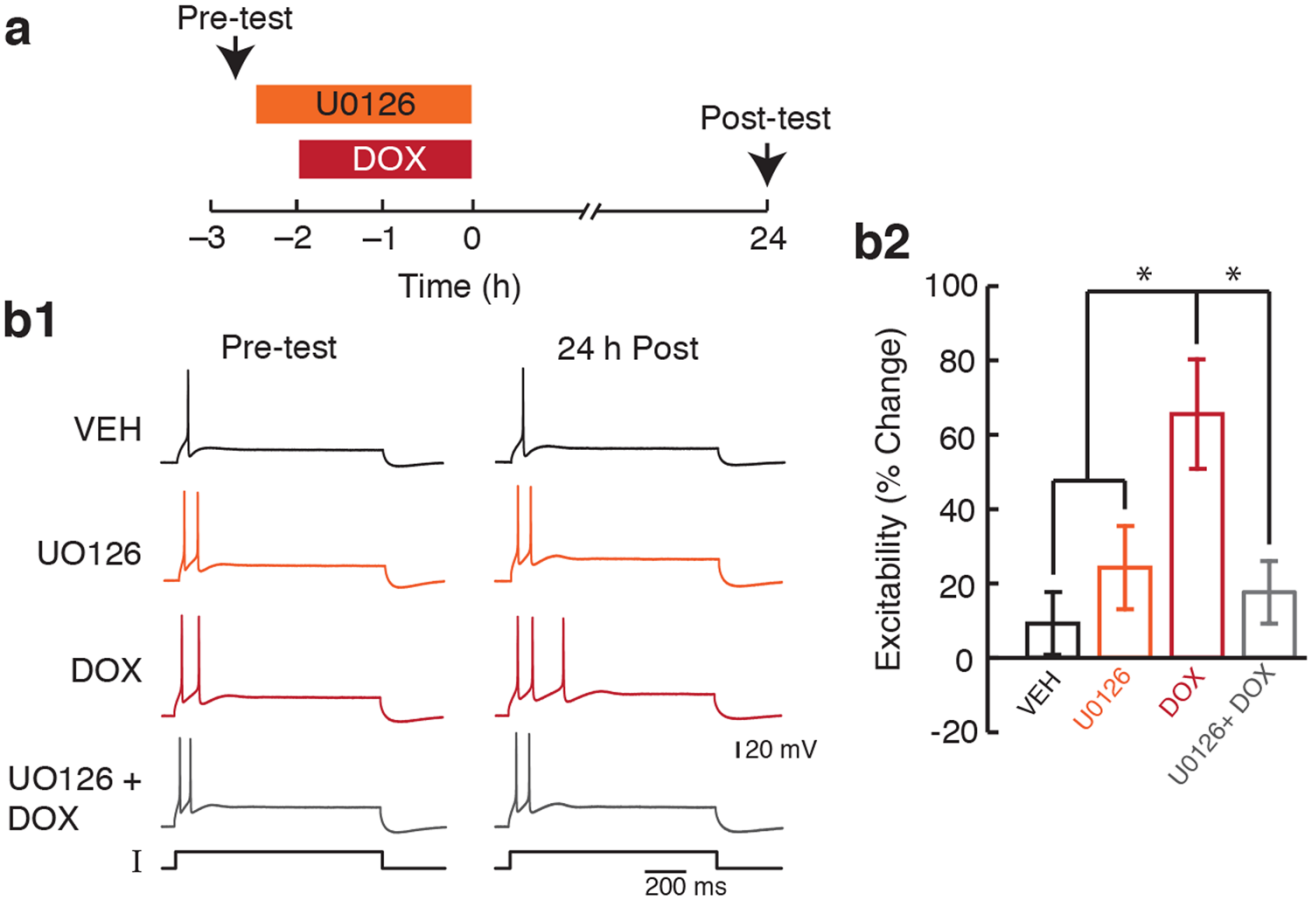
Inhibition of MEK prevented the DOX-induced increase in excitability. (**a**) Protocol for application of U0126 and DOX and measurement of excitability 24 h after the end of treatment. Arrows represent recording times. (**b1**) Representative action potentials recorded before treatment (Pre-test) and 24 h after the end of treatment (24 h Post) in response to a depolarizing current injection (I) into the SN soma. The amplitude of the current injection on both the pre- and post-tests depended upon the pre-test firing threshold (see Materials and Methods for details). In this case, 1 nA was used for all four groups shown. (**b2**) Summary data. The DOX-induced persistent increase in excitability depended on MEK activation. Data are plotted as mean ± SEM; *represents p ≤ 0.05. See also Supplementary Fig. S2 and Supplementary Fig. S3.

Inhibition of MEK either reversed (Fig. 4c) or facilitated (Fig. 4b) the DOX-induced late ERK activation, depending on timing of application of the inhibitor. Given the role of ERK in the induction of SN excitability^31, 34^, it is possible that the DOX-induced persistent increase in SN excitability^12^ (Supplementary Fig. S2) is mediated by ERK activation during DOX treatment. To test this hypothesis, four groups were employed: (1) Veh; (2) U0126; (3) DOX; and (4) U0126 + DOX. DOX led to an increase in excitability of 65.5 ± 14.7% (*n* = 7) but the combination of U0126 + DOX led to only a 17.6 ± 8.4% (*n* = 6) increase in excitability. The changes in excitability induced by Veh and U0126 alone were 9.2 ± 8.4% (*n* = 8) and 24.2 ± 11.2% (*n* = 7), respectively (Fig. 6b). A two-way ANOVA revealed a significant main effect of DOX (F_(1,24)_ = 5.04; p = 0.034), as well as no basal effect of U0126 (F_(1,24)_ = 2.2; p = 0.15). The DOX-induced increase in excitability was blocked by application of U0126 (DOX x U0126; F_(1,24)_ = 8.07; p = 0.009). This result suggests that the DOX-induced long-lasting increase in excitability depends on MEK activation during DOX treatment. In contrast, activation of the p38 MAPK pathway by DOX had no effect on the DOX-mediated increase in excitability (Supplementary Fig. S3).

### Late activation of ERK was negatively correlated with the persistent excitability

**Figure 7:**
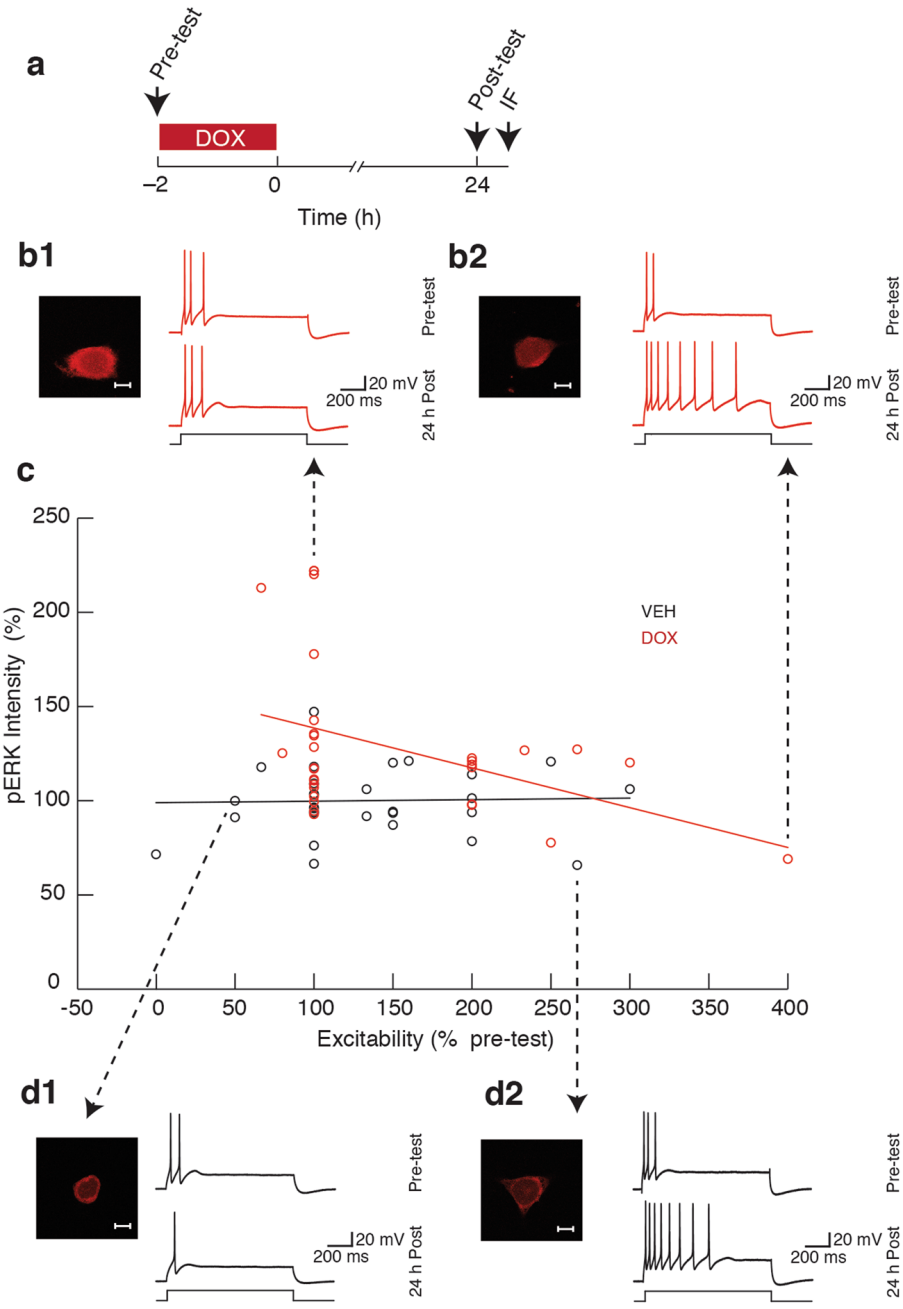
Negative correlation between pERK and excitability 24 h after DOX treatment. (**a**) Protocol for application of DOX and measurement of excitability followed by fixation and IF staining as indicated by arrows. (**b1**) Representative confocal image of a DOX-treated SN (scale bar, 20 µm) and the corresponding action potentials elicited before treatment (Pre-test) and 24 h after the end of treatment (24 h Post) in response to a depolarizing current injection (I) into the SN soma. This SN exhibited a relatively high level of pERK but no change in excitability. (**b2**) Representative confocal image of a DOX-treated SN (scale bar, 20 µm) and corresponding action potentials. This SN exhibited a relatively low level of pERK but enhanced excitability. (**c**) Summary data. Open circles reflect the excitability (x-axis) and the level of pERK (y-axis, represented as percent of Veh) of individual SNs. Black open circles represent individual Veh-treated cells and red open circles represent individual DOX-treated cells. (**d1**) Representative confocal image (scale bar, 20 µm) and corresponding action potentials for a Veh-treated SN that exhibited a decrease in excitability. (**d2**) Representative confocal image (scale bar, 20 µm) and the corresponding action potentials from a Veh-treated SN that exhibited an increase in excitability. pERK and excitability were negatively correlated in DOX-treated SNs but not in Veh-treated SNs. See also Supplementary Fig. S2 and Supplementary Fig. S4.

To understand the relation between the late activation of ERK and the persistent excitability induced by DOX, we determined whether these two phenomena were correlated in individual SNs. Two groups were included in this experiment: (1) Veh; and (2) DOX. SN excitability was measured before and 24 h after treatment, and cells were fixed immediately after the post-test to assess pERK levels (Fig. 7a). Veh-treated cells showed no correlation between excitability and pERK (*n* = 32; r = 0.02; p = 0.9), but DOX-treated cells exhibited a negative correlation between excitability and pERK (*n* = 31; r = 0.41; p = 0.02) (Fig. 7c). Input resistance and pERK were also negatively correlated in DOX-treated cells (Supplementary Fig. S4). Given that the early phase of MEK activation promoted the enhanced excitability, the observed negative correlation between late ERK activation and excitability was unexpected.

## Discussion

**Figure 8:**
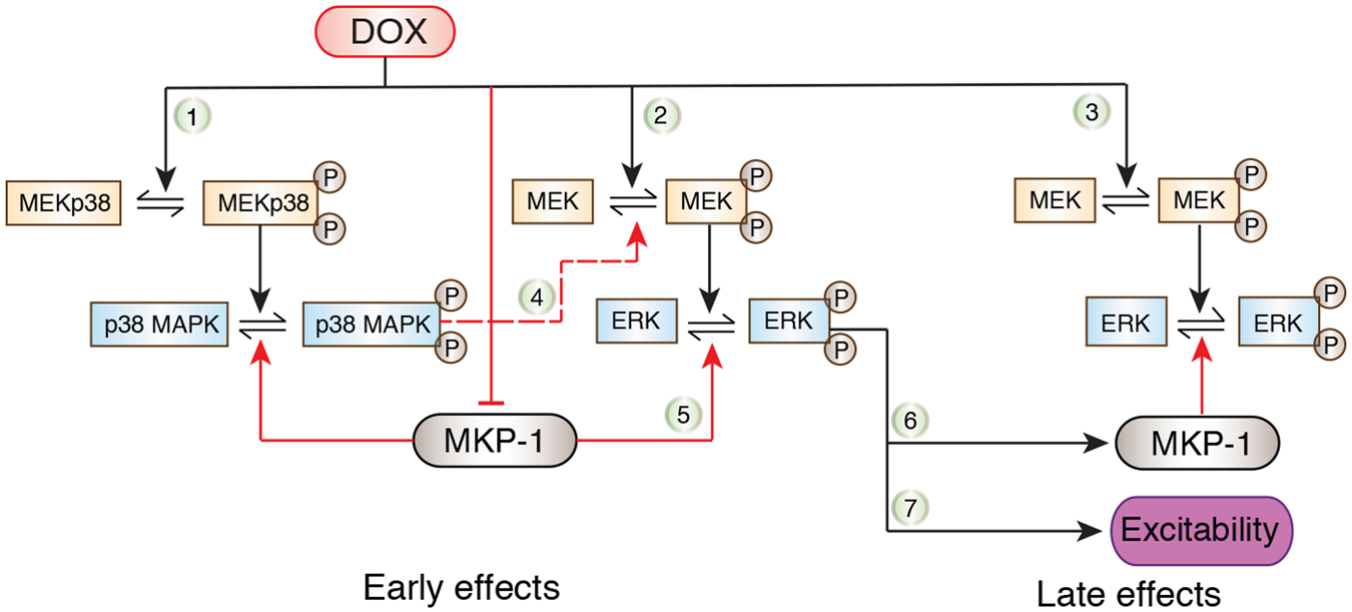
Summary diagram. DOX treatment results in early and late ERK activation, MKP-1 down-regulation and an increase in basal excitability. MEK inhibition during DOX treatment results in a prolonged decrease in MKP-1 expression, potentiation of the late ERK activation reversal of the enhanced excitability. Black solid arrows represent net stimulatory effects whereas red solid arrows and bars represent net inhibitory effects. In the case of reversible reaction arrows, the black solid arrows promote phosphorylation (activation) whereas the red solid arrows promote dephosphorylation (inactivation). Red dotted line represents a putative net inhibitory effect of activated p38 MAPK on activated MEK. Different aspects of DOX-mediated signaling changes are numbered for clarity and ease of reference in the Discussion section.

DOX caused dynamic activation of ERK in neurons with two mechanistically distinct phases and only transient activation of p38 MAPK (Figs 2 and 8, Pathways 1, 2 and 3). ERK and p38 MAPK play opposing roles in the induction of LTF. DOX inhibits LTF in a p38 MAPK-dependent manner despite simultaneous activation of ERK^12^. Therefore, it is logical to expect that DOX may activate p38 MAPK more rapidly than ERK, thereby allowing p38 MAPK to dominate. We found that DOX activated both ERK and p38 MAPK within the first 30 min of exposure (Fig. 1). pERK and p-p38 MAPK remained elevated throughout DOX treatment. Therefore, the dominant effect of p38 MAPK on LTF is likely not due to differences in onset of activation of these two kinases. Nor is it due to persistence of activation of p38 MAPK. One possibility is that DOX-induced p38 MAPK activation may dominate by suppressing activation of the MEK-ERK pathway (Fig. 8, Pathway 4), thus interfering with the dynamics of ERK activity necessary for LTF^20, 25^ in addition to activating cAMP response element-binding protein 2 (CREB2)^12, 18^, which promotes LTD^17, 18^. This inhibitory effect of p38 MAPK on ERK activation (Fig. 8, Pathway 4) in *Aplysia* SNs has been proposed to be a constraint on memory formation^27^ and likely occurs through a phosphatase-dependent mechanism^28^.

Another possibility is that DOX affects processes in the postsynaptic motor neuron (MN) implicated in LTF induction^55^. For example, increased Ca^2+^ in the MN is necessary for LTF induction^56^, and postsynaptic CREB2 has been implicated in the induction of a more persistent form of LTF^57^. Furthermore, some presynaptic changes associated with memory formation are thought to depend on retrograde signals from the postsynaptic MN^55^. Therefore, determining the effects of DOX on the postsynaptic MN would be a logical next step in revealing DOX-dependent changes to the sensorimotor synapse and understanding how DOX may be affecting memory formation.

The kinetics of ERK and p38 MAPK activation in SNs after DOX treatment temporally correlated with the decrease in expression of MKP-1 (Figs 2 and 3). Furthermore, activation of ERK immediately after the end of DOX treatment was not affected by MEK inhibition (Fig. 4a). Thus, the early activation of ERK may reflect the decrease in expression of MKP-1 rather than initial MEK activation (Figs 3, 4a and 8, Pathway 5). However, these results do not exclude the possibility that DOX does activate MEK at the start of the treatment, but accumulation of p-p38 MAPK and the resulting inhibition masks this effect (Fig. 8, Pathway 4). Indeed, inhibition of MEK during DOX treatment had multiple effects including blockade of the enhanced excitability (Figs 4b, 5b and 6) suggesting that the MEK-ERK pathway is activated during DOX treatment (Fig. 8, Pathway 2).

The DOX-induced late ERK activation was, at least in part, dependent on delayed MEK activation (Figs 4c and 8, Pathway 3) and likely not attributable to a late decrease in MKP-1 levels as MKP-1 protein returned to basal levels soon after the end of DOX treatment (Fig. 3). The late activation of ERK occurs when activated p38 MAPK levels are no longer elevated (Fig. 2) and thus may function to promote normal synaptic facilitation. The late ERK activation was potentiated by MEK inhibition during DOX treatment (Fig. 4b). Because MEK inhibition during DOX treatment also prolonged the decrease in levels of MKP-1 protein (Fig. 5b), it is possible that this decrease in MKP-1 was the cause of the enhanced DOX-induced late ERK activation. Furthermore, the ability of a MEK inhibitor to decrease expression of MKP-1 suggests that the MEK-ERK pathway promotes expression of MKP-1 protein in SNs (Fig. 8, Pathway 6). The MEK-ERK pathway can modulate MKP-1 protein expression through both enhanced transcription^51–54^ as well as stabilization of MKP-1 protein through decreased ubiquitin-proteasome-dependent degradation^47^. However, at 24 h, DOX treatment by itself does not alter MKP-1 levels even though levels of ERK activation are increased. Therefore, MEK inhibition modulates the effects of DOX treatment on pERK, but DOX stimulation of MKP-1 expression is evident only when unmasked by MEK inhibition.

MEK-mediated ERK activation occurring before accumulation of p-p38 MAPK and the consequent inhibition of MEK, may be sufficient to induce long-lasting effects such as the persistent increase in basal excitability^12^ (Supplementary Fig. S2, Figs 6 and 8, Pathway 7). Consistent with the enhancement of basal excitability, DOX also increased the input resistance and decreased the firing threshold of SNs (Supplementary Fig. S2). Together, these changes promote an increase in firing probability upon stimulation. These biophysical alterations caused by DOX are similar to changes observed during memory formation^58^, but DOX prevents 5-HT-mediated increases in SN excitability^12^, suggesting occlusion. Furthermore, inhibition of MEK, not p38 MAPK, reversed the DOX-mediated increase in basal excitability (Fig. 6 and Supplementary Fig. S3), whereas inhibition of p38 MAPK rescued DOX-induced impairment of LTF^12^. Thus, whereas the effects of DOX-mediated activation of p38 MAPK are dominant over ERK in the case of synaptic plasticity (i.e., LTF and LTD), basal excitability is unaffected by p38 MAPK activation.

The late ERK activation and enhanced basal excitability were negatively correlated in individual neurons (Fig. 7c). Furthermore, MEK inhibition both blocked the DOX-induced enhanced excitability and potentiated the late DOX-induced ERK activation (Figs 4b and 6). These results raise questions about the underlying mechanisms and whether they are linked﻿﻿: 1) One possibility is a homeostatic mechanism in which the late ERK activation tends to reverse the DOX-induced enhanced excitability. This may be advantageous given that deleterious side-effects of DOX can be reversed by reducing neuronal activity^15^. Thus, the early increase in ERK and p38 MAPK activity may be associated with a decrease in the level of MKP-1 while the late MEK-mediated increase in ERK activation levels may be an adaptive mechanism to counteract the enhanced excitability. Such a cascade of events can potentially explain the biphasic activation of ERK (Fig. 2). Alternatively, the late excitability or some aspect of its underlying mechanism may feedback to lower the level of pERK to suppress aberrant changes in excitability. A finding of this nature would be represented in the pathway diagram (Fig. 8) as a net inhibition leading from Excitability to the late pERK. 2) The negative correlation could also be due to a feedforward mechanism and be related to the early levels of ERK activation. Individual SNs containing higher levels of pERK during DOX treatment may go on to exhibit higher levels of excitability and higher levels of MKP-1 at later time points which would promote dephosphorylation of ERK. Conversely, SNs containing lower levels of pERK initially may show lower levels of excitability and MKP-1 protein later, and therefore relatively higher levels of pERK. Such an effect would explain the negative correlation between pERK and excitability 24 h after DOX treatment.

Investigations into these emerging hypotheses would contribute significantly to our understanding of how DOX affects neurons and mechanisms implicated in memory formation in addition to possibly uncovering protective mechanisms employed by neurons in response to chemotherapy that includes DOX. A possibility that needs to be considered is that in *Aplysia*, U0126 may be less specific, resulting in off-target effects such as modulation of p38 MAPK or other kinases. The same idea applies to SB 203580. Furthermore, given the known role of DOX in inducing oxidative damage^1^, it is important to determine the extent to which the effects of DOX on the signaling pathways and the physiological changes associated with memory formation are caused by oxidative damage.

## Materials and Methods

### Sensory neuronal cultures

Primary cell cultures were prepared using SNs from the pleural ventrocaudal cluster of the marine mollusk *Aplysia californica* according to established procedures^31^. *Aplysia* (60–100 g) were purchased from the US National Institutes of Health *Aplysia* resource facility (University of Miami). Each culture dish contained 5–10 SNs, and SNs were incubated in culture medium (50% L15, 50% hemolymph) for 4–6 days.

### Pharmacological treatment

To examine the time course of DOX-induced pERK and p-p38 MAPK in SNs, DOX (2.5 µM, Sigma) or Veh solution containing 50% L15 medium and 50% artificial seawater (ASW, comprised of 450 mM NaCl, 10 mM KCl, 11 mM CaCl_2_, 29 mM MgCl_2_, 10 mM HEPES at pH 7.6) was applied for 30, 60, 90, and 120 min. DOX concentrations used *in vitro* vary widely (0.5 to 20 μM)^12, 45, 46, 59, 60^. However, *in vivo* measurement of the amount of DOX in rodent brain after intraperitoneal injection is approximately 0.25 μM^11^. Similar effects on kinase activation are observed in cortical neurons at both concentrations of DOX (0.25 μM and 2.5 μM)^12^. SNs were then fixed immediately after treatment and processed for immunofluorescence analyses. To examine the persistent effects of DOX (2.5 µM, 2 h), DOX was washed out at the end of treatment and SNs were subsequently incubated in Veh solution for the 1- and 2-h time-points. For the 24- and 48-h time-points, cells were incubated in culture medium until the respective times of fixation. For each experiment, DOX-treated SNs were normalized to Veh-treated SNs.

To determine whether MEK or p38 MAPK activation during DOX treatment contributes to the DOX-induced increase in basal excitability or long-lasting ERK activation, SNs were incubated with U0126 (20 µM in 0.2% DMSO, Promega) or SB 203580 (3 µM in 0.2% DMSO, EMD Millipore), respectively, 30 min prior to and during DOX treatment (2.5 µM, 2 h). U0126 (20 µM) decreases pERK levels in *Aplysia* SNs^61^, and SB 203580 inhibits the activity of *Aplysia* p38 MAPK *in vitro* ^18^. The concentration of SB 203580 chosen inhibits FMRFa-induced LTD but has no basal effects on synaptic strength^18^. SNs were subsequently incubated in culture medium and excitability was tested or cells were fixed 24 h after the end of treatment. To determine whether the late ERK activation depends on late MEK activation, U0126 was applied to SNs 21.5 h after the end of DOX treatment and cells were fixed 2.5 h later (24 h after DOX).

### Immunofluorescence

SNs were processed for immunofluorescence as described^12, 31^. SNs were fixed using 4% paraformaldehyde (wt/vol) in phosphate-buffered saline (PBS) containing 30% sucrose for 20 min (4 °C) and then rinsed twice with PBS. Subsequently, cells were blocked for 30 min (room temperature) in Superblock buffer (Pierce), 0.2% Triton X-100, and 3% normal goat serum followed by overnight incubation (4 °C) with anti-pERK antibody (1:200, Cell Signaling Technology), anti-p-p38 MAPK antibody (1:200, Cell Signaling Technology) or anti-MKP -1 antibody (1:500, Santa Cruz Biotechnology). Cells were rinsed twice with PBS and then incubated with secondary antibody (goat anti-rabbit IgG conjugated to Cy-3; 1:200 dilution, Jackson Laboratory) for 1 h (room temperature). Cells were rinsed, mounted on coverslips, and images of sections located in the middle of the nuclei were obtained with a confocal microscope (Zeiss LSM510) using a 63x oil-immersion lens as described^12^. Mean fluorescence intensity was measured with MetaMorph software (version 7.5, Molecular Devices). Intensity measurements from at least four neurons on each coverslip were averaged. The levels of pERK or p-p38 MAPK were normalized to levels measured in Veh-treated SNs. Consequently, sample sizes reported in the Results represent number of coverslips.

These, and all subsequent experiments were performed in a blind manner such that the experimenter was unaware of the treatment group identities until the end of the analyses.

### Electrophysiology

Biophysical properties of cultured SNs were measured as previously described^12^. SNs were impaled with sharp microelectrodes (10–20 MΩ resistance) that were filled with 3 M potassium acetate and attached to an Axoclamp 2-B amplifier (Molecular Devices) Data were acquired and analyzed with the aid of pClamp software (version 10.2, Molecular Devices). SNs were current clamped at −45 mV (for a few early experiments) or −55 mV, and 1 s of hyperpolarizing current (0.3 nA) was injected to assess input resistance. Next, depolarizing current was injected in increasing 0.1-nA steps (1 s each) until an action potential was triggered. Firing threshold was recorded as the lowest current intensity required to elicit an action potential. The magnitude of depolarizing current used to evaluate excitability depended on the firing threshold. When the firing threshold was between 0.1 and 0.4 nA, 0.5 nA of depolarizing current was injected and when the threshold was between 0.5 and 0.9 nA, 1.0 nA of depolarizing current was injected. Excitability was calculated as a percent change compared to the number of action potentials elicited at pretest. Each data point represents a mean percent change for all SNs in a single dish. Individual SNs were excluded from use at pretest if their firing threshold was less than or equal to 0.2 nA or if their resting membrane potential was more depolarized than −40 mV. Individual SNs were excluded from analyses if their input resistance decreased by 50% or more from the pre- to post-test or if their resting membrane potential was more depolarized than −30 mV on the post-test. For Fig. 6, Supplementary Fig. S2, and Supplementary Fig. S3, only experiments containing a Veh-treated group that did not show a large increase in excitability (dish average of 50% or more) between the pre- and post-test were included for analysis. Responses from 2–5 neurons per dish were averaged. Consequently, sample sizes reported for Fig. 6, Supplementary Fig. S2, and Supplementary Fig. S3 represent number of dishes. For the correlation analyses in Fig. 7 and Supplementary Fig. S4, data points represent individual SNs, and so cells were included regardless of the magnitude of the average response from all cells in the dish. Consequently, sample sizes reported for Fig. 7 and Supplementary Fig. S4 represent number of cells.

### Correlation

To examine the correlation between pERK levels and biophysical properties 24 h after DOX treatment, properties were measured before and 24 h after DOX treatment as indicated previously. Immediately after the 24 h post-test, SNs were fixed and stained with anti-pERK antibody (1:200) and then processed for immunofluorescence analysis. SNs examined during the biophysical measurements were identified and imaged to determine pERK levels.

### Statistical analyses

Matlab (version 8.3; Mathworks) and Sigmaplot (version 12; Systat Software) were used to plot and analyze data. Two-way ANOVAs followed by either Tukey’s or Fisher’s least significant difference (LSD) were used in experiments involving multiple comparisons. Student’s paired and unpaired t-tests were used for comparisons between two groups. Correlation coefficients were calculated as Pearson’s; exact sample sizes are included in the text. All data passed tests of normality and equal variances. Data are presented as means ± SEM; p ≤ 0.05 was considered to represent statistical significance.

## Electronic supplementary material

Supplementary Information

## References

[1] Ahles TA, Saykin AJ (2007). Candidate mechanisms for chemotherapy-induced cognitive changes. Nature Rev. Can..

[2] Myers JS (2009). Chemotherapy-related cognitive impairment. Clin J. of Oncol. Nurs..

[3] Tannock, I. F., Ahles, T. A., Ganz, P. A. & van Dam, F. S. Cognitive impairment associated with chemotherapy for cancer: report of a workshop. *J. Clin. Oncol.* **22**, 2233–2239 (2004). 10.1200/JCO.2004.08.094 15169812

[4] Wefel JS, Saleeba AK, Buzdar AU, Meyers CA (2010). Acute and late onset cognitive dysfunction associated with chemotherapy in women with breast cancer. Cancer..

[5] Christie LA (2012). Impaired cognitive function and hippocampal neurogenesis following cancer chemotherapy. Clin. Cancer Res..

[6] Liedke PE (2009). Systemic administration of doxorubicin impairs aversively motivated memory in rats. Pharmacol. Biochem. Behav..

[7] Seigers R (2015). Cognitive impact of cytotoxic agents in mice. Psychopharmacology..

[8] Shan K, Lincoff AM, Young JB (1996). Anthracycline-induced cardiotoxicity. Ann. Intern. Med..

[9] Raj S, Franco VI, Lipshultz SE (2014). Anthracycline-induced cardiotoxicity: a review of pathophysiology, diagnosis, and treatment. Curr. Treat. Options Cardiovasc. Med..

[10] Nhan T, Burgess A, Lilge L, Hynynen K (2014). Modeling localized delivery of Doxorubicin to the brain following focused ultrasound enhanced blood-brain barrier permeability. Phys. Med. Biol..

[11] Sardi I (2013). Pharmacological modulation of blood-brain barrier increases permeability of doxorubicin into the rat brain. Am. J. Cancer Res..

[12] Liu RY, Zhang Y, Coughlin BL, Cleary LJ, Byrne JH (2014). Doxorubicin attenuates serotonin-induced long-term synaptic facilitation by phosphorylation of p38 mitogen-activated protein kinase. J. Neurosci..

[13] Salas-Ramirez KY (2015). Doxorubicin and cyclophosphamide induce cognitive dysfunction and activate the ERK and AKT signaling pathways. Beh. Brain Res..

[14] Cleary LJ, Lee WL, Byrne JH (1998). Cellular correlates of long-term sensitization in. Aplysia. J. Neurosci..

[15] Moruno Manchon JF (2016). Levetiracetam mitigates doxorubicin-induced DNA and synaptic damage in neurons. Sci. Rep..

[16] Bolshakov VY, Carboni L, Cobb MH, Siegelbaum SA, Belardetti F (2000). Dual MAP kinase pathways mediate opposing forms of long-term plasticity at CA3-CA1 synapses. Nat. Neurosci..

[17] Guan Z (2002). Integration of long-term-memory-related synaptic plasticity involves bidirectional regulation of gene expression and chromatin structure. Cell..

[18] Guan Z (2003). p38 MAP kinase mediates both short-term and long-term synaptic depression in *Aplysia*. J. Neurosci..

[19] Liang YC, Huang CC, Hsu KS (2008). A role of p38 mitogen-activated protein kinase in adenosine A(1) receptor-mediated synaptic depotentiation in area CA1 of the rat hippocampus. Mol. Brain..

[20] Martin KC (1997). MAP kinase translocates into the nucleus of the presynaptic cell and is required for long-term facilitation in *Aplysia*. Neuron..

[21] Michael D (1998). Repeated pulses of serotonin required for long-term facilitation activate mitogen-activated protein kinase in sensory neurons of *Aplysia*. Proc. Natl. Acad. Sci. USA.

[22] Murray HJ, O’Connor JJ (2003). A role for COX-2 and p38 mitogen activated protein kinase in long-term depression in the rat dentate gyrus *in vitro*. Neuropharmacology..

[23] Ormond J (2004). ApTrkl, a Trk-like receptor, mediates serotonin-dependent ERK activation and long-term facilitation in *Aplysia* sensory neurons. Neuron..

[24] Purcell AL, Sharma SK, Bagnall MW, Sutton MA, Carew TJ (2003). Activation of a tyrosine kinase-MAPK cascade enhances the induction of long-term synaptic facilitation and long-term memory in *Aplysia*. Neuron..

[25] Sharma SK, Carew TJ (2004). The roles of MAPK cascades in synaptic plasticity and memory in *Aplysia*: facilitatory effects and inhibitory constraints. Learn. Mem..

[26] Sweatt JD (2004). Mitogen-activated protein kinases in synaptic plasticity and memory. Curr. Opin. Neurobiol..

[27] Fioravante D, Smolen PD, Byrne JH (2006). The 5-HT- and FMRFa-activated signaling pathways interact at the level of the Erk MAPK cascade: potential inhibitory constraints on memory formation. Neurosci. Lett..

[28] Junttila MR, Li SP, Westermarck J (2008). Phosphatase-mediated crosstalk between MAPK signaling pathways in the regulation of cell survival. FASEB J..

[29] Li SP, Junttila MR, Han J, Kähäri VM, Westermarck J (2003). p38 Mitogen-activated protein kinase pathway suppresses cell survival by inducing dephosphorylation of mitogen-activated protein/extracellular signal-regulated kinase kinase1, 2. Can. Res..

[30] Adams JP, Sweatt JD (2002). Molecular psychology: roles for the ERK MAP kinase cascade in memory. Annu. Rev. Pharmacol. Toxicol..

[31] Chin J, Liu RY, Cleary LJ, Eskin A, Byrne JH (2006). TGF-β-induced long-term changes in neuronal excitability in *Aplysia* sensory neurons depend on MAPK. J. Neurophysiol..

[32] Cohen-Matsliah SI, Brosh I, Rosenblum K, Barkai E (2007). A novel role for extracellular signal-regulated kinase in maintaining long-term memory-relevant excitability changes. J. Neurosci..

[33] Rosenkranz JA, Frick A, Johnston D (2009). Kinase-dependent modification of dendritic excitability after long-term potentiation. J. Physiol..

[34] Sung YJ, Povelones M, Ambron RT (2001). RISK-1: a novel MAPK homologue in axoplasm that is activated and retrogradely transported after nerve injury. J. Neurobiol..

[35] Poolos NP, Bullis JB, Roth MK (2006). Modulation of h-Channels in hippocampal pyramidal neurons by p38 mitogen-activated protein kinase. J. Neurosci..

[36] Ster J (2007). Exchange protein activated by cAMP (Epac) mediates cAMP activation of p38 MAPK and modulation of Ca2+ -dependent K+ channels in cerebellar neurons. Proc. Natl. Acad. Sci. USA.

[37] Hudmon A (2008). Phosphorylation of Sodium Channel Nav1.8 by p38 Mitogen-Activated Protein Kinase Increases Current Density in Dorsal Root Ganglion Neurons. J. Neurosci..

[38] Jin X, Gereau RW (2006). Acute p38-mediated modulation of tetrodotoxin-resistant sodium channels in mouse sensory neurons by tumor necrosis factor-alpha. J. Neurosci..

[39] Zhang R (2010). Acute p38-mediated inhibition of NMDA-induced outward currents in hippocampal CA1 neurons by interleukin-1β. Neurobiol. Dis..

[40] Camps M, Nichols A, Arkinstall S (2000). Dual specificity phosphatases: a gene family for control of MAP kinase function. FASEB J..

[41] Michael D (1998). Repeated pulses of serotonin required for long-term facilitation activate mitogen-activated protein kinase in sensory neurons of *Aplysia*. Proc. Natl. Acad. Sci. USA.

[42] Jeffrey KL, Camps M, Rommel C, Mackay CR (2007). Targeting dual-specificity phosphatases: manipulating MAP kinase signalling and immune responses. Nat. Rev. Drug Discov..

[43] Liu Y, Shepherd EG, Nelin LD (2007). MAPK phosphatases–regulating the immune response. Nat. Rev. Immunol..

[44] Wancket LM, Frazier WJ, Liu Y (2012). Mitogen-activated protein kinase phosphatase (MKP)-1 in immunology, physiology, and disease. Life Sci..

[45] Rojo F (2009). Mitogen-activated protein kinase phosphatase-1 in human breast cancer independently predicts prognosis and is repressed by doxorubicin. Clin. Cancer Res..

[46] Small GW, Somasundaram S, Moore DT, Shi YY, Orlowski RZ (2003). Repression of mitogen-activated protein kinase (MAPK) phosphatase-1 by anthracyclines contributes to their antiapoptotic activation of p44/42-MAPK. J. Pharmacol. Exp. Ther..

[47] Small GW (2004). Evidence that mitogen-activated protein kinase phosphatase-1 induction by proteasome inhibitors plays an antiapoptotic role. Mol. Pharmacol..

[48] Choi J (2008). The effect of doxorubicin on MEK-ERK signaling predicts its efficacy in HCC. J. Surg. Res..

[49] Fey D, Croucher DR, Kolch W, Kholodenko BN (2012). Crosstalk and signaling switches in mitogen-activated protein kinase cascades. Front. Physiol..

[50] Smolen P, Baxter DA, Byrne JH (2008). Bistable MAPK activity: A plausible mechanism contributing to maintenance of late long-term potentiation. Am. J. Physiol..

[51] Brondello JM, Brunet A, Pouysségur J, McKenzie FR (1997). The dual specificity mitogen-activated protein kinase phosphatase-1 and -2 are induced by the p42/p44MAPK cascade. J. Biol. Chem..

[52] Caunt CJ, Keyse SM (2013). Dual-specificity MAP kinase phosphatases (MKPs): shaping the outcome of MAP kinase signalling. FEBS J..

[53] Cook SJ, Beltman J, Cadwallader KA, McMahon M, McCormick F (1997). Regulation of mitogen-activated protein kinase phosphatase-1 expression by extracellular signal-related kinase-dependent and Ca2+ -dependent signal pathways in Rat-1 cells. J. Biol. Chem..

[54] Li J (2001). Transcriptional induction of MKP-1 in response to stress is associated with histone H3 phosphorylation–acetylation. Mol. Cell Biol..

[55] Glanzman DL (2008). New tricks for an old slug: the critical role of postsynaptic mechanisms in learning and memory in. Aplysia. Prog. Brain Res..

[56] Cai D, Chen S, Glanzman DL (2008). Postsynaptic regulation of long-term facilitation in. Aplysia. Curr. Biol..

[57] Hu JY, Levine A, Sung YJ, Schacher S (2015). cJun and CREB2 in the postsynaptic neuron contribute to persistent long-term facilitation at a behaviorally relevant synapse. J. Neurosci..

[58] Mozzachiodi R, Byrne JH (2010). More than synaptic plasticity: role of nonsynaptic plasticity in learning and memory. Trends Neurosci..

[59] Poizat C, Puri PL, Bai Y, Kedes L (2005). Phosphorylation-dependent degradation of p300 by doxorubicin-activated p38 mitogen-activated protein kinase in cardiac cells. Mol. Cell. Biol..

[60] Price, D. M., Wloka, M. T., Chik, C. L. & Ho, A. K. Mitogen‐activated protein kinase phosphatase‐1 (MKP‐1) preferentially dephosphorylates p42/44MAPK but not p38MAPK in rat pinealocytes. *J. Neurochem.* **101**, 1685–1693 (2007). 10.1111/j.1471-4159.2007.04557.x 17437549

[61] Chin, J., Angers, A., Cleary, L. J., Eskin, A. & Byrne, J. H. Transforming growth factor β1 alters synapsin distribution and modulates synaptic depression in *Aplysia. J. Neurosci.* **22**, 1–6 (2002). 10.1523/JNEUROSCI.22-09-j0004.2002 PMC6758346 11978861

