# Supplementary material for: Characterization and reversal of Doxorubicin-mediated biphasic activation of ERK and persistent excitability in sensory neurons of Aplysia californica
Source: Sci Rep. 2017 Jul 3;7:4533. doi: 10.1038/s41598-017-04634-4 (PMC5495788; doi:10.1038/s41598-017-04634-4)
Supplement: Supplementary file 1 — Supplementary Information [file 41598_2017_4634_MOESM1_ESM.pdf]

Supplementary Information

Characterization and reversal of Doxorubicin-mediated biphasic activation of ERK and persistent excitability in sensory neurons of *Aplysia californica*

Harini Lakshminarasimhan[1,3,+], Brittany L. Coughlin[1,4,+], Amber S. Darr[2], John H. Byrne[1,4,*]

[1] Department of Neurobiology and Anatomy

W.M. Keck Center for the Neurobiology of Learning and Memory

McGovern Medical School at The University of Texas Health Science Center at Houston

6431 Fannin St, Suite MSB 7.046

Houston, Texas 77030, USA

[2] McGovern Medical School at The University of Texas Health Science Center at Houston

6431 Fannin St, Suite MSB 7.046

Houston, Texas 77030, USA

[3] Present address: Department of Physiology, Feinberg School of Medicine, Northwestern University

303 E Superior Street, Chicago, Illinois 60611

[4] The University of Texas MD Anderson Cancer Center UTHealth Graduate School of Biomedical Sciences, Houston, TX

6767 Bertner Ave, Mitchell Bldg. BSRB S3.8344

Houston, Texas 77030, USA

[+] These authors contributed equally to this work

[*] Corresponding author: J. H. Byrne, John.H.Byrne@uth.tmc.edu

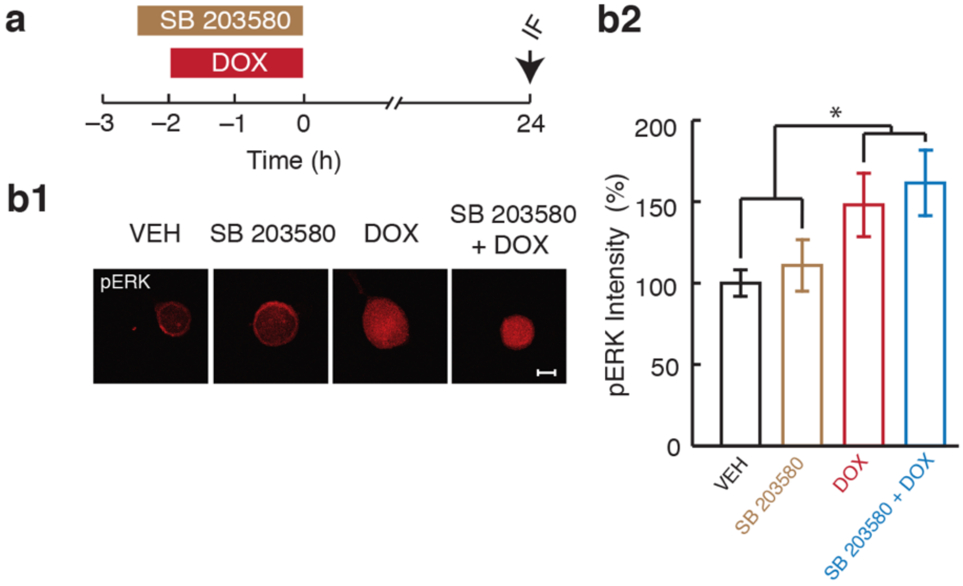

Figure S1. Inhibition of p38 MAPK during DOX treatment does not affect the late ERK activation. (**a**) Protocol for application of SB 203580 and DOX followed by measurement of ERK activation 24 h after the end of treatment. Arrow represents fixation time and subsequent IF staining. (**b1**) Representative confocal images of pERK staining in SNs 24 h after the end of DOX treatment. Scale bar, 20 μm. (**b2**) Summary data. DOX induced late activation of ERK independent of p38 MAPK. A two-way ANOVA revealed a significant main effect of DOX on pERK ($F_{(1,56)} = 8.8$; $p = 0.004$) as well as no basal effect of SB 203580 on pERK ($F_{(1,56)} = 0.54$; $p = 0.4$). The DOX-induced increase in pERK was unaffected by application of SB 203580 (DOX x SB 203580; $F_{(1,56)} = 0.006$; $p = 0.9$). DOX and SB 203580 + DOX treated groups were significantly different from the Veh-treated groups ($p = 0.045$ and $p = 0.01$, respectively). Data are plotted as mean ± SEM; * represents $p \leq 0.05$.

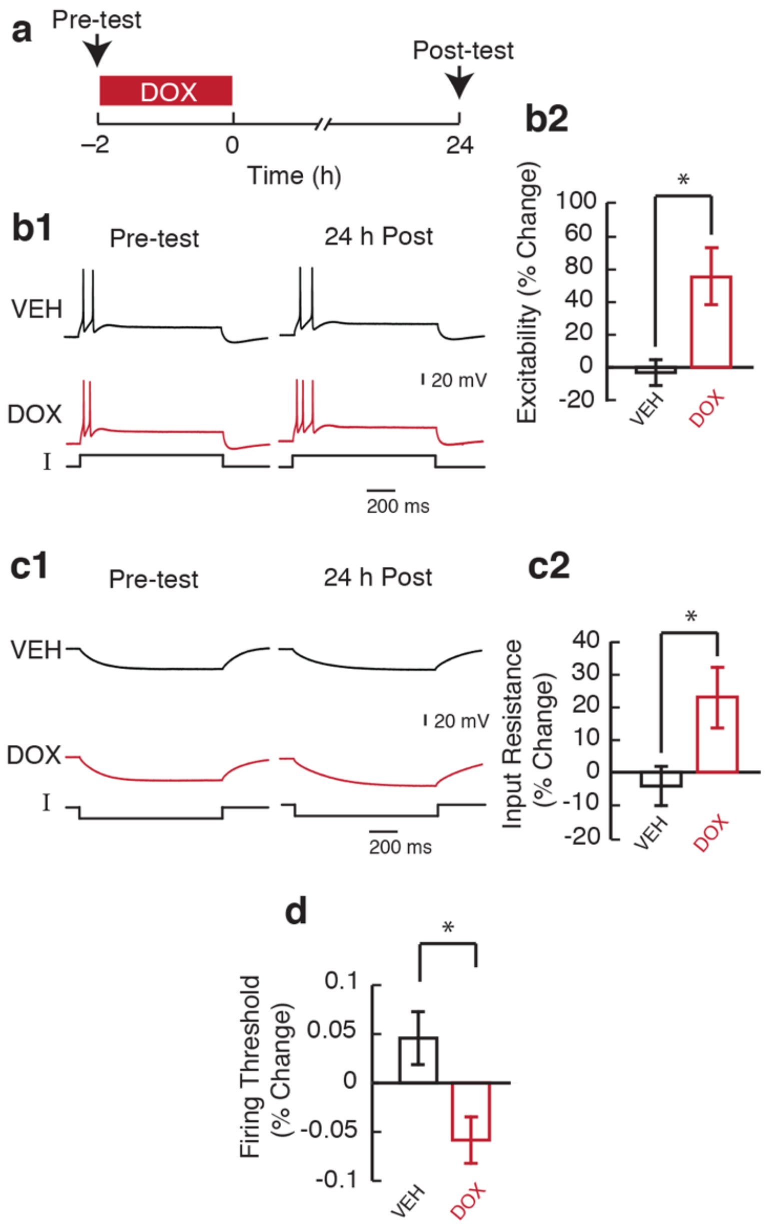

Figure S2. DOX induced persistent increases in excitability and input resistance. (**a**) Protocol for application of DOX and measurement of biophysical properties. Arrows represent recording times. (**b1**) Representative action potentials recorded before treatment (pre-test), and 24 h (24 h post) after the end of treatment in response to a depolarizing current injection (I) into the SN soma. The amplitude of the current injection on both the pre- and post-tests depended upon the pre-test firing threshold (see Materials and Methods for details). In this case, the amplitude was 1 nA for both the Veh and DOX traces. (**b2**) Summary data. DOX treatment enhanced basal excitability of SNs ($t_{(18)}$ = 3.1;

p = 0.006). (**c1**) Representative traces showing input resistance before treatment (pre-test), and 24 h (24 h post) after the end of treatment in response to a 0.3 nA hyperpolarizing current injection (I) into the SN soma. (**c2**) Summary data. DOX treatment increased input resistance of SNs ($t_{(18)}$ = 2.5; p = 0.02). (**d**) Firing threshold summary data. DOX treatment decreased firing threshold of SNs ($t_{(18)}$ = 2.9; p = 0.01). Data are plotted as mean ± SEM; * represents p ≤ 0.05.

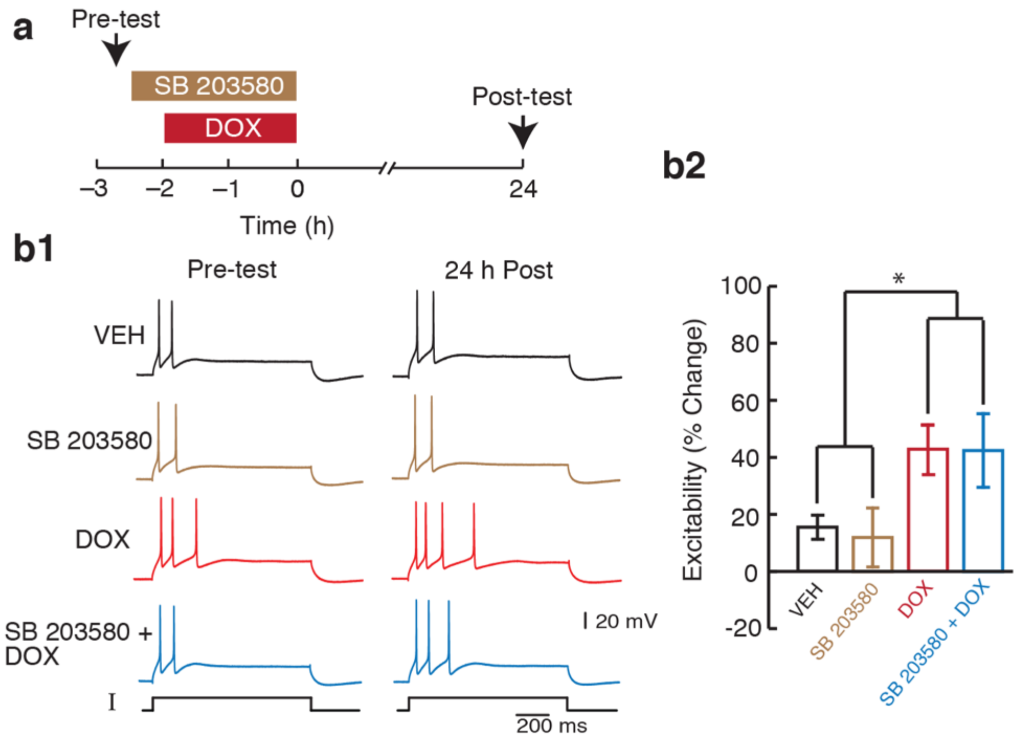

Figure S3. Inhibition of p38 MAPK did not affect DOX-induced persistent increase in excitability. (**a**) Protocol for application of SB 203580 and DOX and measurement of excitability 24 h after treatment. Arrows represent recording times. (**b1**) Representative action potentials recorded before treatment (pre-test) and 24 h after the end of treatment (24 h post) in response to a depolarizing current injection (I) into the SN soma. The amplitude of the current injection on both the pre- and post-tests depended upon the pre-test firing threshold (see Materials and Methods for details). In this case, the amplitude was 0.5 nA for all groups. (**b2**) Summary data. DOX induced a persistent increase in excitability independent of p38 MAPK. A two-way ANOVA revealed a significant main effect of DOX treatment on excitability ($F_{(1,39)} = 11.0$; $p = 0.002$) as well as no basal effect of SB 203580 on excitability ($F_{(1,39)} = 0.05$; $p = 0.82$). The DOX-induced increase in excitability was unaffected by application of SB 203580 (DOX x SB 203580; $F_{(1,39)} = 0.03$; $p = 0.86$). DOX and SB 203580 + DOX treated groups were significantly different from the Veh-treated groups ($p = 0.02$ and $p = 0.04$, respectively). Data are plotted as mean ± SEM; * represents $p \leq 0.05$.

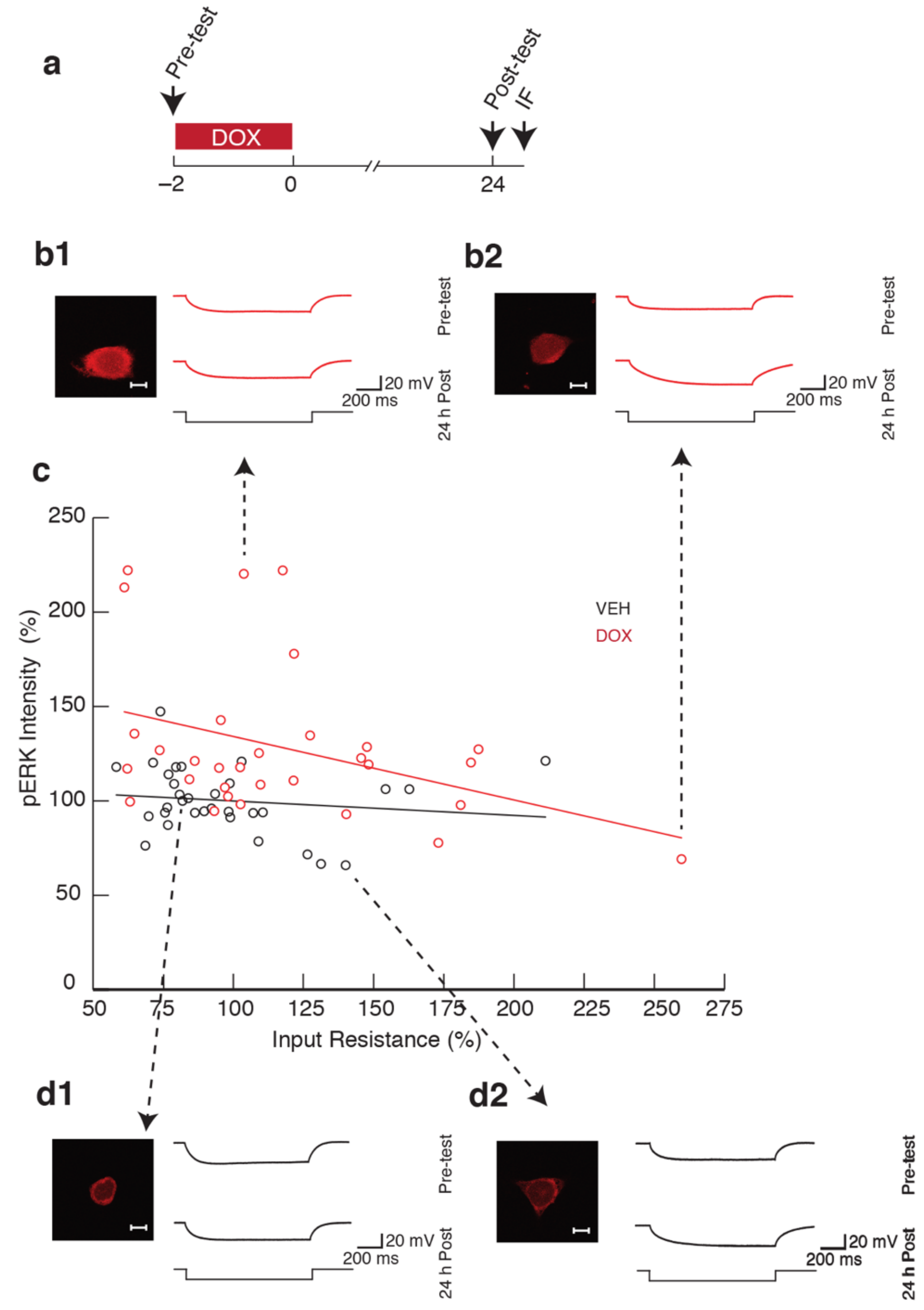

Figure S4. Negative correlation between pERK and input resistance in SNs 24 h after DOX treatment. (**a**) Protocol for application of DOX and measurement of input resistance followed by fixation and IF staining as indicated by arrows. (**b1**) Representative

confocal image of a DOX-treated SN (scale bar, 20 μm) and the corresponding traces elicited by a 0.3 nA hyperpolarizing current injection before treatment (Pre-test) and 24 h after the end of treatment (24 h Post). This SN exhibited a relatively high level of pERK but no change in input resistance. (**b2**) Representative confocal image of a DOX-treated SN (scale bar, 20 μm) and corresponding traces. This SN exhibited a relatively low level of pERK but an increase in input resistance. (**c**) Summary data. Veh-treated cells showed no correlation between input resistance and pERK (*n* = 32; r = 0.14; p = 0.44), but DOX-treated cells exhibited a negative correlation between input resistance and pERK levels (*n* = 31; r = 0.37; p = 0.04). Open circles represent input resistance (x-axis, represented as percent of pre-test) and level of pERK (y-axis, represented as percent of Veh) of individual SNs. Black open circles represent individual Veh-treated cells and red open circles represent individual DOX-treated cells. (**d1**) Representative confocal image (scale bar, 20 μm) and corresponding traces for a Veh-treated SN that exhibited a decrease in input resistance. (**d2**) Representative confocal image (scale bar, 20 μm) and the corresponding traces from a Veh-treated SN that exhibited an increase in input resistance.
